# Evaluation of different total *Leishmania amazonensis* antigens for the development of a first-generation vaccine formulated with a Toll-like receptor-3 agonist to prevent cutaneous leishmaniasis

**DOI:** 10.1590/0074-02760200067

**Published:** 2020-07-13

**Authors:** María José Germanó, Esteban Sebastián Lozano, María Victoria Sanchez, Flavia Alejandra Bruna, María Fernanda García-Bustos, Arianna Lourdes Sosa Lochedino, María Cristina Salomón, Ana Paula Fernandes, Juan Pablo Mackern-Oberti, Diego Esteban Cargnelutti

**Affiliations:** 1 Consejo Nacional de Investigaciones Científicas y Tecnológicas, Universidad Nacional de Cuyo, Instituto de Medicina y Biología Experimental de Cuyo, Mendoza, Argentina; 2 Universidad Nacional de Cuyo, Facultad de Ciencias Médicas, Mendoza, Argentina; 3 Consejo Nacional de Investigaciones Científicas y Tecnológicas, Instituto de Patología Experimental, Salta, Argentina; 4 Universidade Federal de Minas Gerais, Faculdade de Farmácia, Belo Horizonte, MG, Brasil.

**Keywords:** L. amazonensis, whole-cell vaccine, Poly (I:C), leishmaniasis

## Abstract

**BACKGROUND:**

Unfortunately, no any vaccine against leishmaniasis has been developed for human use. Therefore, a vaccine based on total *Leishmania* antigens could be a good and economic approach; and there are different methodologies to obtain these antigens. However, it is unknown whether the method to obtain the antigens affects the integrity and immune response caused by them.

**OBJECTIVES:**

to compare the protein profile and immune response generated by total *L. amazonensis* antigens (TLA) produced by different methods, as well as to analyse the immune response and protection by a first-generation vaccine formulated with sonicated TLA (sTLA) and polyinosinic:polycytidylic acid [Poly (I:C)].

**METHODS:**

TLA were obtained by four different methodologies and their integrity and immune response were evaluated. Finally, sTLA was formulated with Poly (I:C) and their protective immune response was measured.

**FINDINGS:**

sTLA presented a conserved protein profile and induced a strong immune response. In addition, Poly (I:C) improved the immune response generated by sTLA. Finally, sTLA + Poly (I:C) formulation provided partial protection against *L. amazonensis* infection.

**MAIN CONCLUSIONS:**

The protein profile and immune response depend on the methodology used to obtain the antigens. Also, the formulation sTLA + Poly (I:C) provides partial protection against cutaneous leishmaniasis in mice.

Leishmaniasis is an anthropozoonotic disease caused by intracellular protozoa belonging to the *Leishmania* genus. It is transmitted between animals and humans by the bite of an infected Phlebotomine female. This disease is considered a growing public health problem as a consequence of the tropicalisation of continents.^1^ It is also considered a neglected disease by the World Health Organization, due to the fact that it mainly affects low income populations with limited access to health care.

Different clinical manifestation can be caused by different species of *Leishmania*: cutaneous, mucocutaneous and visceral leishmaniasis. However, cutaneous leishmaniasis can be presented as localised, diffuse and atypical.^2^ Particularly, *L. amazonensis* causes localised cutaneous leishmaniasis. Nevertheless, this specie has been isolated from patients with diffuse cutaneous and mucocutaneous leishmaniasis,^3^ and some cases of visceral leishmaniasis by *L. amazonensis* have been described in humans and dogs.^4^

Currently, there is not any approved vaccine against human leishmaniasis.^5^ Nevertheless, several vaccine formulations are under evaluation in preclinical^6^ ^,^ ^7^ ^,^ ^8^ and clinical trials (ClinicalTrials.gov Identifier: NCT02894008 and NCT03969134). The need to induce a Th1-type immune response in order to obtain protection was shown in *Leishmania*-murine models protection studies and in the immune profiles of self-healed individuals.^9^

First-generation *Leishmania* vaccines containing total *Leishmania* antigens are proposed as an economic strategy to prevent leishmaniasis,^10^ which is an important point in low income countries, where this disease is endemic. In this type of vaccine formulations, antigens have been produced by different methodologies based on the promastigote form: cycles of freezing and thawing, ultrasound, autoclaving, and pasteurisation.^11^ ^,^ ^12^ ^,^ ^13^ ^,^ ^14^ Notably, only this kind of vaccines against human leishmaniasis has been evaluated in phase III clinical trials.^15^

Few studies have been reported about the antigenic profile of total *Leishmania* antigens.^13^ ^,^ ^16^ To date, no study has been conducted for the comparative assessment of the immune response generated by total *Leishmania* antigens produced by different methodologies.

The development of new vaccine strategies should be based on recent knowledge of the innate immune response, which can be activated by molecular patterns associated with pathogens through the activation of Toll-like receptors (TLRs). This activation has an important role in the direction of the acquired immune response. TLRs agonists have shown promising therapeutic and prophylactic effects in disease models. It is important to remark that TLR-4 and TLR-9 agonists have been approved to be used as adjuvants in human vaccines.^17^ We recently reported that a synthetic double-stranded RNA TLR-3 agonist [Poly (I:C)] formulated with freezing and thawing TLA triggers a protective immune response.^6^ There is a lot of evidence that demonstrates the capacity of Poly (I:C) to induce an activation of antigen presentation cells (APC), a Th1-like immune response as well as generate memory T and B cells.^18^

In the present study, we evaluated the antigenic profile and the immune response generated by TLA obtained from promastigotes by cycles of freezing and thawing (fTLA), ultrasound (sTLA), autoclaving (aTLA), and pasteurisation (pTLA). After that, we selected the methodology that generated a more conserved antigenic profile and the antigens that generated greater immune response, to be formulated with a TLR-3 agonist. Finally, we evaluated the capacity of this formulation to mediate a Th1-like immune response and to provide protective immunity against *L. amazonensis* infection in BALB/c mice.

## MATERIALS AND METHODS

*Parasite cultures and methodologies used to obtain TLA* - *L. amazonensis* promastigotes (MHOM/VE/84/MEL) were grown in NNN (Novy-MacNeal-Nicolle) medium and their infectivity was maintained by serial passages in mice, as described previously.^19^

The TLA were obtained from *L. amazonensis* promastigotes, which were grown at their late logarithmic phase; afterwards, they were harvested by centrifugation and washed three times with phosphate-buffered saline (PBS). Four different methodologies were used to disrupt the promastigotes: one cycle of autoclaving at 121ºC for 15 min (aTLA);^11^ five cycles of freezing (-80ºC) and thawing (56ºC) (fTLA);^12^ one cycle of pasteurisation at 56ºC for 30 min (pTLA);^13^ and one cycle of ultrasound at 40 W for 1 min at 4ºC (sTLA).^14^ The protein content of each TLA was measured by bicinchoninic acid assay following manufacturing instructions (Thermo-Scientific).

*Analysis of TLA protein profile* - Sixty µg of each TLA and promastigote culture (fresh) were separated and analysed by electrophoresis in 12.5% sodium dodecyl sulfate-polyacrylamide gel electrophoresis (SDS-PAGE) under reducing conditions and stained with Coomasie Blue R-250.

Densitometric analysis and molecular weight determinations of each TLA protein profile were performed by Fiji ImageJ software version 2.0 [US National Institutes of Health (NIH); http://rsb.info.nih.gov/ij/].

Finally, the level of bacterial endotoxin was determined using the Gel Clot LAL Assay (Associates of Cape Cod, Inc. Falmouth, MA, USA), at the microbiological unit of Microquim Inc, Contract Scientific Research Laboratory (Buenos Aires, Argentina).

*Animals* - Inbred female BALB/c mice (six-to-eight-week old) were used in this study. Mice were kept in standard conditions with barriers as well as controlled light cycle and temperature. Food and water were provided *ad libitum*. Two independent experiments were carried out.

*Ethics* - All animals were cared for in accordance with the Guiding Principles in the Care and Use of Animals of the US NIH. All procedures performed in studies involving animals were under the ethical standards and were approved by the Institutional Animal Care and Use Committee of the School of Medical Science, Universidad Nacional de Cuyo (protocol approval no. 80/2016).

*Immunisation scheme* - In order to compare the immune response of animals to each TLA, four animals per group were injected twice every 21 days with 100 µg of aTLA, fTLA, pTLA or sTLA without adjuvant by subcutaneous route into the interscapular zone; whereas the control group received PBS.

In order to evaluate the immune response and protection generated by a first-generation vaccine, 12 animals per group were injected three times every 14 days with: PBS (control); 100 µg of sTLA alone or with 50 µg of high molecular weight Poly (I:C) (Invivogen).

*Humoral immune response* - Anti-TLA total IgG, IgG1 and IgG2a subclasses were measured by enzyme-linked immunosorbent assay (ELISA), as previously described.^19^ Data are represented as the mean ± standard error of mean (SEM) of optical density (OD) values of the vaccinated group.

Cellular immune response

*In vitro culture cells* - Two weeks after their last immunisation, animals were sacrificed and their spleens were removed and homogenised in supplemented RPMI-1640 medium (Gibco) containing 10% foetal bovine serum (FBS), 100 IU/mL penicillin, 100 mg/mL streptomycin and β-mercaptoethanol. Red blood cells were disrupted using ammonium-chloride-potassium lysing buffer. Splenocytes were incubated in triplicate at 5 x 10^5^ cells per well into 96-wells-flat-bottom culture plates at 37ºC in 5% CO_2_ and stimulated for 72 h with 1 µg of the same TLA to the one used for immunisation or supplemented medium alone.^11^

*Cytokines measurement* - Levels of IFN-γ, IL-4 and IL-10 cytokines were determined in the supernatant of stimulated splenocytes by OptEIA ELISA Kits according to manufacturing instructions (BD Pharmingen). Values are represented as means ± SEM of pg/mL of each cytokine.

*In vitro cell proliferation* - To determine cell proliferation after 72 h of stimulation,^20^ splenocytes were incubated with 0.5 mg/mL of Thiazolyl Blue Tetrazolium Bromide (MTT, Sigma) for 4 h. After discarding the supernatants, formazan crystals from cells were dissolved with 100 µL of dymethil sulphoxide (DMSO). The absorbance was read at 570 nm and the proliferation index (PI) was calculated according to the following formula:

PI = (OD value from stimulated cell) 

 (OD value from non-stimulated cell)

Non-stimulated cells from each group were used as control for proliferation. Results are represented as means ± SEM of proliferation index (ratio of unstimulated control).

*Parasite challenge and infection progression* - Six animals per group immunised with PBS, sTLA and sTLA + Poly (I:C) were infected on the right footpad (RFP) with 1 x 10^4^ *L. amazonensis* promastigotes at their stationary phase.^21^ The swelling of RFP was measured weekly using digital caliper (SCHWYZ, ED-10P) until 11 weeks after infection. The value for uninfected footpads was subtracted from each infected footpad to estimate the lesion size. Results were represented as mm of footpad swelling.

*Splenic index* - The splenic index was used as an indicator of parasite visceralisation. Eleven weeks after challenge, mice were weighted, sacrificed and their spleens removed and weighted to calculate splenic index according to the following formula:

Splenic index = (Spleen weight)

(Body weight) * 100

*Determination of parasite burden by limit dilution* - Parasite burden was determined by limit dilution 11 weeks after challenge following a protocol described.^22^ Briefly, mice were sacrificed and their infected footpads were removed and homogenised in 1 mL of supplemented RMPI 1640 medium. The suspension was plated in 96-wells plate and 10-fold dilutions were made and incubated at 26ºC for 14 days in order to assess viable parasites under optic microscopy. The wells containing motile promastigotes were identified and the number of viable parasites was determined from the highest dilution at which promastigotes had grown. The results were represented as -Log number of parasite.

*Statistical analysis* - In order to establish statistically significant differences, the data were analysed by analysis of variance (ANOVA), using Graphpad prism software version 5.00 (La Jolla California USA, www.graphpad.com).

## RESULTS

**Fig. 1:**
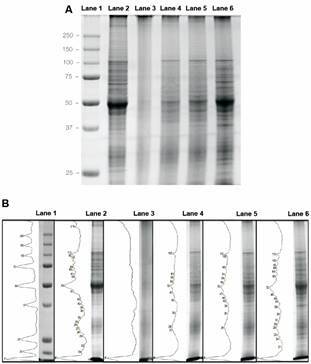
protein profile of total *Leishmania amazonensis* antigens (TLA). Sixty μg of total protein from fresh (lane 2), autoclaving TLA (aTLA) (lane 3), freezing and thawing TLA (fTLA) (lane 4), pasteurisation TLA (pTLA) (lane 5) and sonicated TLA (sTLA) (lane 6) were analysed by electrophoresis on a sodium dodecyl sulfate-polyacrylamide gel electrophoresis (SDS-PAGE) (12.5%). Molecular weight markers are shown in lane 1, with corresponding kDa on the left. The gel was stained with Coomassie blue (A). Densitometric analysis and MW determination in kDa (B).

*sTLA has a conserved protein profile* - The SDS-PAGE profiles from fresh promastigotes, aTLA, fTLA, pTLA, and sTLA are shown in Fig. 1A. The integrity of proteins in sTLA and fresh extracts have notable similarity in the intensity and relative mobility of protein profile, widely distributed in a range of 25 to 250 kDa. Conversely, patterns of aTLA, fTLA, pTLA are clearly distinct with a smaller number of conserved bands. In addition, a densitometric analysis and molecular weight (MW) quantification of protein profile were performed, with results presented in kDa (Fig. 1B). A more intense band of 52 kDa was observed mainly in fresh and sTLA.

The endotoxin concentration of each TLA was less than 500 EU/mL. The recommendation of < 500 EU/mL for inactivated vaccines is based on the complex nature of these vaccines containing multiple antigens.^23^

*sTLA induces a strong humoral immune response* - Animals were injected twice at 3-week intervals with PBS (control), aTLA, fTLA, pTLA, and sTLA by subcutaneous route to determine if the methodology employed to obtain TLA could induce differential immune response. Specific anti-*Leishmania* IgG and its subclasses, IgG1 and IgG2a, were measured by ELISA two weeks after boost.

**Fig. 2:**
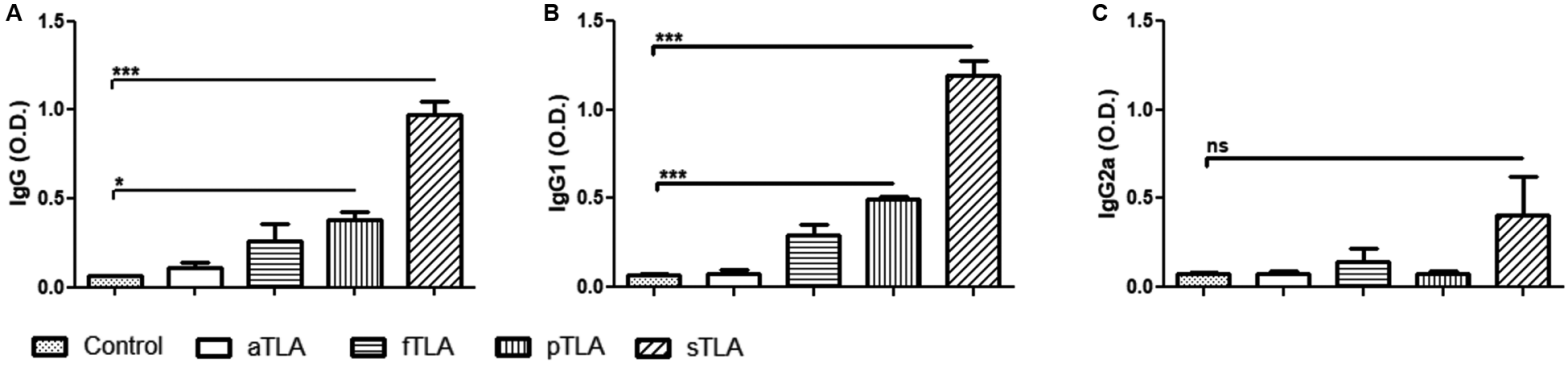
humoral immune response in mice injected with different total *Leishmania amazonensis* antigens (TLA). Anti-*Leishmania* IgG antibodies (A) using serum samples dilution (1:500) obtained two weeks after boost. Anti-*Leishmania* IgG1 (B) and IgG2a (C) antibodies using serum samples dilution (1:1000) obtained two weeks after boost. Results are represented as mean ± standard error of mean (SEM) of optical density (OD) values obtained from four animals per groups. Asterisks indicate significant differences in comparison to the control group: *p ˂ 0.05; ***p ˂ 0.001; ns: not significant by analysis of variance (ANOVA).

Mice that received sTLA showed higher IgG levels (p < 0.001), followed by mice injected with pTLA (p < 0.05), while aTLA and fTLA did not show statistically significant increase in comparison to the control group (Fig. 2A).

Furthermore, mice injected with sTLA showed the highest IgG1 levels (p < 0.001) followed by mice that received pTLA (p < 0.05). In spite of the fact that IgG2a levels were not significant for all groups, sTLA showed a tendency to reach higher levels than other experimental groups (Fig. 2B, C).

**Fig. 3:**
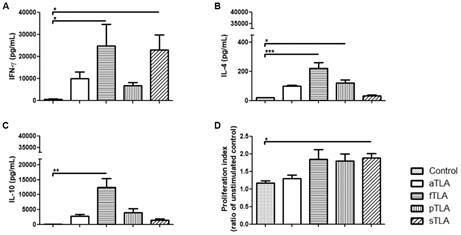
cellular immune response generated in mice injected with different total *Leishmania amazonensis* antigens (TLA). Cytokines levels of IFN-γ (A), IL-4 (B), IL-10 (C) measured by enzyme-linked immunosorbent assay (ELISA) using supernatant of splenocytes stimulated with TLA two weeks after boost; results are represented as mean ± standard error of mean (SEM) of cytokine concentration (pg). Cell proliferation assay (D) determined by MTT on stimulated splenocytes two weeks after boost; results are represented as mean ± SEM of proliferation index (ratio of unstimulated control). Asterisks indicate significant differences in comparison to the control group: *p ˂ 0.05; **p ˂ 0.01; ***p ˂ 0.001.

*sTLA increased IFN-γ levels and cell proliferation* - We performed antigen-specific stimulatory spleen cells cultures from immunised mice to determine whether TLA-responder cells secrete Th polarised cytokines. As it can be seen in Fig. 3, both fTLA and sTLA induced an intense IFN-γ production in contrast with the control group (p ˂ 0.05). When IL-4 production was determined, fTLA and pTLA displayed higher levels than the control group (p ˂ 0.001 and p ˂ 0.05). Finally, only fTLA induced a marked increase in IL-10 production (p ˂ 0.01). These results suggest that fTLA displayed a striking reactivity but rising different Th profiles such as Th1, Th2 and Tr1. In contrast, sTLA induced an increase of IFN-γ, with low IL-4 and IL-10 levels. In addition, fTLA, pTLA and sTLA induced cell proliferation; however, only sTLA displayed a statistically significant increase compared with the control group (Fig. 3D).

Altogether, these data highly suggest that sTLA displayed higher IFN-γ level and cellular proliferation, with lower IL-10 and lL-4 levels than the other TLA being eligible to perform a T cell-mediated vaccine.

*sTLA formulated with Poly (I:C) enhances humoral immune responses* - In order to improve the immune response toward a Th1 profile, sTLA was formulated with Poly (I:C) as adjuvant. Mice were immunised three times at 2-week intervals with PBS (control), sTLA and sTLA + Poly (I:C) and antibody levels were measured.

**Fig. 4:**
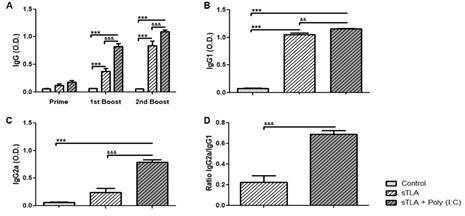
humoral immune response in mice immunised with sonicated total *Leishmania amazonensis* antigens (sTLA) + Poly (I:C). Anti-*Leishmania* IgG antibodies (A) using serum samples dilution (1:500) obtained two weeks after each immunisation. Anti-*Leishmania* IgG1 (B) and IgG2a (C) antibodies using serum samples dilution (1:500) obtained two weeks after boost. IgG2a/IgG1 ratio (C). Results are represented as mean ± standard error of mean (SEM) of optical density (OD) values obtained from six mice per groups. Asterisks indicate significant differences in comparison to the control group: ***p ˂ 0.001. ^&^ indicates statistically significant differences compared with the sTLA group: ^&&^p < 0.01; ^&&&^p < 0.001.

After the first boost, vaccine formulation containing sTLA and sTLA + Poly (I:C) induced an statistically significant increase of IgG antibodies in comparison to control group (p < 0.001). However, immunisation with sTLA + Poly (I:C) induced higher IgG levels than sTLA alone (p < 0.001) (Fig. 4A).

In addition, mice immunised with sTLA + Poly (I:C) showed significantly higher IgG1 levels than sTLA alone (p < 0.01) as well as the control group (p < 0.001) (Fig. 4B). It was remarkable that mice immunised with sTLA + Poly (I:C) showed significantly superior IgG2a levels as compared to sTLA alone and the control group (p < 0.001) (Fig. 4C). In agreement with this, the IgG2a/IgG1 ratio was higher in the sTLA + Poly (I:C) formulation (Fig. 4D). In conclusion, the combination of sTLA with Poly (I:C) induced a strong and Th1/Th2 balanced humoral immune response.

**Fig. 5:**
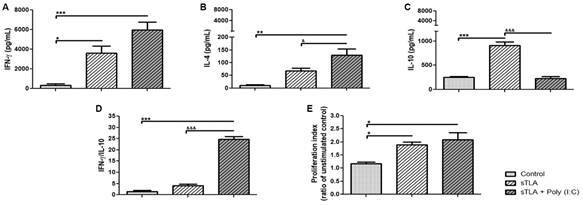
cellular immune response generated in mice immunised with sonicated total *Leishmania amazonensis* antigens (sTLA) + Poly (I:C). Cytokines levels of IFN-γ (A), IL-4 (B), IL-10 (C) and IFN-γ / IL-10 ratio (D) were measured by enzyme-linked immunosorbent assay (ELISA) using supernatant of splenocytes stimulated with sTLA two weeks after the last boost; results are represented as mean ± standard error of mean (SEM) of cytokine concentration (pg) from six animals per group. Cell proliferation assay (E) was determined by MTT on stimulated splenocytes two weeks after the last boost; results are represented as mean ± SEM of proliferation index (ratio of unstimulated control) from six animals per group. Asterisks indicate significant differences in comparison to the control group: *p ˂ 0.05; **p ˂ 0.01; ***p ˂ 0.001. ^&^ indicates statistically significant differences compared with the sTLA group: ^&^p < 0.05; ^&&&^p < 0.001.

*sTLA formulated with Poly (I:C) induces a strong Th1-like cellular immune response* - Two weeks after the last boost, cytokines production and cell proliferation were analysed on splenocyte culture. As can be seen in Fig. 5, sTLA + Poly (I:C) generated higher IFN-γ production than sTLA as compared to the control group (p < 0.001 and p < 0.05). Additionally, Poly (I:C) also increased sTLA´s ability to secrete IL-4 (p < 0.05). Furthermore, sTLA + Poly (I:C) produced significantly lower IL-10 levels than sTLA group (p < 0.001). Although both sTLA and sTLA + Poly (I:C) were able to induce significant cell proliferation in stimulated spleen cells (p < 0.05), sTLA + Poly (I:C) formulation had a tendency to reinforce this response (Fig. 5E).

These dataset highly indicated a Th1 bias cellular response induced by the sTLA + Poly (I:C) immunisation.

**Fig. 6:**
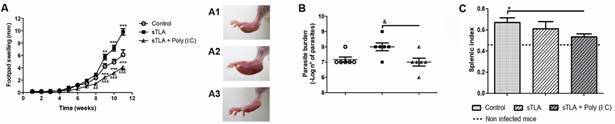
protection assay on immunised mice immunised with sonicated total *Leishmania amazonensis* antigens (sTLA) + Poly (I:C). Footpad swelling caused by challenge with infective promastigotes of *L. amazonensis* (A), and photograph of right footpad (RFP) taken nine weeks after infection from control (A1), sTLA (A2) and sTLA + Poly (I:C) (A3) groups. Parasite burden in infected footpads, the numbers of viable parasites were determined after 11 weeks of infection by a limit dilution assay (B). Splenic index was evaluated 11 weeks after infection (C). Values in the graphics represent the means ± standard error of mean (SEM) of footpad swelling (mm), parasite burden (-Log n° of parasites) or splenic index from 6 animals per group. The asterisks indicate statistically significant differences compared with the control group: *p < 0.05; **p < 0.01; ***p < 0.001. ^&^ indicates statistically significant differences compared with the sTLA group: ^&^p < 0.05; ^&&^p < 0.01; ^&&&^p < 0.001.

*sTLA formulated with Poly (I:C) provides partial protection against L. amazonensis infection* - To determine the effectiveness of the immune response generated by a first-generation vaccine formulated with sTLA and Poly (I:C), different protection parameters were analysed: footpad swelling, splenic index and parasite burden (Fig. 6). Mice immunised with sTLA + Poly (I:C) showed a significant reduction in the size of their footpad lesion as compared to the control mice (p < 0.001 since nine weeks after challenge) or the group receiving sTLA in the absence of adjuvant (p < 0.01 since eight weeks after infection).

Furthermore, sTLA alone showed a significant increase in their footpad swelling compared with control group (p < 0.01 since nine weeks after infection). The differences in the size of footpad lesion could be noted macroscopically in the week nine after infection (Fig. 6A1, A2, A3).

Parasite burden and splenic index were determined 11 weeks after infection. Mice immunised with sTLA + Poly (I:C) showed a significant reduction of parasite burden in comparison to the group receiving sTLA alone (p < 0.05) but showed no difference in comparison to the control group (Fig. 6B). The splenic index of mice vaccinated with sTLA + Poly (I:C) was similar to non-infected mice, exhibiting a more significant reduction than the control group (p < 0.05), which had an intense splenomegaly (Fig. 6C).

## DISCUSSION

The research and development of vaccines against leishmaniasis began in the 20th century with first-generation vaccines comprising total *Leishmania* antigens. In the new world, Mayrink and Convit were the pioneers in the development of these prophylactic strategies, who produced total *Leishmania* antigens by ultrasound and pasteurisation to be combined with different adjuvant. These formulations showed to be immunogenic, safe and effective against cutaneous and/or mucocutaneous leishmaniasis.^13^ ^,^ ^24^ In the 21st century, third generation vaccines were developed and evaluated, on the basis of recombinant proteins or DNA vectors.^25^ Two veterinary vaccines against leishmaniasis based on recombinant antigens are already licensed.^26^ Although many vaccine antigens have been identified in attempts to develop a *Leishmania* vaccine, only first-generation vaccines succeeded to reach phase III clinical trials.^15^

Even though different methodologies are used to obtain total *Leishmania* antigens for the development of first generation vaccines, no many studies have been conducted to compare the protein profile and immune response produced by these *Leishmania* antigens.^16^

Our study shows that the protein pattern of aTLA had a great loss of integrity and it was similar to that observed in other studies.^13^ In concordance, the humoral and cellular immune response induced by aTLA was very low. Otherwise, fTLA presented a conserved protein profile with a Th1:Th2:Tr1 mixed immune response; this was also observed in our previous reports.^6^ The comparison between protein integrity of pTLA and aTLA was similar to the one observed by Convit et al.^13^ Finally, the integrity of proteins in ultrasound and fresh extracts had a notable similarity: protein profiles were essentially unaltered. In concordance, sTLA produced a strong humoral immune response.

Although splenocytes proliferation measurement in response to antigenic stimulation is not a direct correlation of a specific effector function of T lymphocytes, it is reliable and widely used to assess the overall immunogenic features of antigenic proteins.^27^ Our results demonstrate that only stimulated splenocytes from mice injected with sTLA had a statistically significant increase of cell proliferation. Thus, the increase in the cell proliferation index and the type of produced cytokines indicate that sTLA activates a cellular immune response characterised by higher levels of IFN-γ and cellular proliferation than control (Fig. 3). However, it was not enough to protect against *L. amazonensis* infection, due to those immunised with sTLA developed a high increase of footpad swelling and parasite load respect to control group (Fig. 6).

We previously demonstrated that subcutaneous vaccination with fTLA without an adjuvant increases the susceptibility of BALB/c mice to cutaneous leishmaniasis.^6^ ^,^ ^19^ Therefore, TLA induce an exacerbation of the infection independently of the methodology employed to obtain them. These results were in accordance to other authors, who demonstrated that whole-killed promastigotes of *L. amazonensis* administrated to Rhesus monkeys via a subcutaneous route also showed an increase in experimental infections with follow up challenges with *L. amazonensis*.^28^ This increased susceptibility to cutaneous leishmaniasis could be due to the presence of serine proteases in whole-killed promastigotes of *L. amazonensis*, which promote an enhanced production of IL-10 and activate a Th2-type immune response.^29^

In spite of the strong immune response induced by sTLA, it must be reinforced towards a better Th1-like immune response by an appropriated adjuvant. Our results disclose that Poly (I:C) improved the humoral immune response of sTLA due to the adjuvant increased IgG2a levels and IgG2a/IgG1 ratio with respect to sTLA alone (Fig. 4).

Cytokine production and cell proliferation are different methodologies to evaluate the cellular immune response mediated by T-lymphocytes.^30^ Our results demonstrate that sTLA + Poly (I:C) enhanced this response, increasing IFN-γ production and cell proliferation. Even though this formulation also increased IL-4 production, it was lower than IFN-γ and it was accompanied by basal levels of IL-10; and it correlated with an increase of ratio IFN-γ/IL-10. It has been demonstrated that IL-10 has a significant role in infection progression,^31^ so maintaining low levels of IL-10 implies an improvement in the protective immune response.

Our previous results showed that Poly (I:C) formulated with TLA, obtained by cycles of freezing and thawing, was able to protect BALB/c mice against *L. amazonensis* infection.^6^ The present study confirms that Poly (I:C) combined with another TLA, obtained by ultrasound, also provides partial protection against cutaneous leishmaniasis. Protective response was reflected in a lower footpad lesion, splenic index and parasite load; however, it must be improved. The reduction in splenic index could be indirectly related to a decrease in parasite visceralisation,^32^ an inflammatory responses and to a lower susceptibility to infection.^33^ Despite of the most conserved protein profile of sTLA, our previous studies with fTLA demonstrated an apparent higher protection, with lower footpad swelling. However, a comparative experiment should be conducted.

In conclusion, the antigens obtained by ultrasound have a more conserved protein profile and induce an intense immune response in immunised animals. These antigens formulated with a synthetic double-stranded RNA as adjuvant lead to an increase of IgG2a and IFN-γ, accompanied by basal levels of IL-10. These datasets highly suggest that sTLA + Poly (I:C) is an eligible formulation to generate a balanced Th1/Th2 humoral and Th1 cellular immune response that allows a partial protection against cutaneous leishmaniasis by *L. amazonensis*.
